# A secondary multivariate decomposition analysis of factors influencing smokeless tobacco cessation across two Global Adult Tobacco Survey waves in India and Bangladesh

**DOI:** 10.18332/tpc/215183

**Published:** 2026-01-29

**Authors:** Shalini Bassi, Manu Raj Mathur, Monika Arora, Tina Rawal, Rajmohan Panda, Ali Golkari

**Affiliations:** 1 Public Health Foundation of India, New Delhi, India; 2 Centre for Dental Public Health and Primary Care, Institute of Dentistry, Queen Mary University of London, London, United Kingdom

**Keywords:** smokeless tobacco, cessation, health knowledge, attitudes, media exposure

## Abstract

**INTRODUCTION:**

This study explores patterns and determinants of attempted quitting of smokeless tobacco (AQSLT) in India and Bangladesh, using data from two waves of the Global Adult Tobacco Survey (GATS).

**METHODS:**

A secondary analysis was conducted using nationally representative data from GATS Wave 1 (2009–2010) and Wave 2 (2016–2017) in India and Bangladesh. Adults who reported using SLT currently or within 12 months at the time of each wave were included. Changes in AQSLT prevalence, associated factors, and contribution of independent variables were assessed using descriptive statistics, logistic regression, and multivariate decomposition analysis.

**RESULTS:**

While smokeless tobacco (SLT) use declined from Wave 1 to Wave 2 in both countries, changes in AQSLT prevalence were not statistically significant. In India, multivariate decomposition revealed that increases in the proportion of individuals who received quitting advice from a doctor or healthcare professional, noticed health warnings on SLT products, saw SLT warnings in print media, and were exposed to pro-SLT advertisements, had positive endowment effects on AQSLT. Behavioral changes among those exposed to SLT product warnings had the strongest negative composition effect. In Bangladesh, increased exposure to warnings in print media had positive, while decreased exposure to pro-SLT advertisements had a negative endowment effect on AQSLT. No significant composition effects were observed in Bangladesh.

**CONCLUSIONS:**

Interventions such as providing advice and health warnings show inconsistent effects on quit behavior. Findings warrant further evaluation of effectiveness of interventions and exploring tested culturally sensitive cessation strategies, that effectively motivate quit attempts among SLT users.

## INTRODUCTION

Smokeless tobacco (SLT) is a major contributor to the global tobacco burden, with over 300 million people using SLT products worldwide^1^. The World Health Organization’s Framework Convention on Tobacco Control (FCTC) defines smokeless tobacco as ‘tobacco that is consumed in unburnt form either orally or nasally’^2^. SLT use is associated with a wide range of adverse health outcomes, including oral, oesophageal, and pancreatic cancers, as well as an increased risk of heart disease and stroke^3^. Globally, SLT use contributes to over 0.65 million deaths annually^4^. While global tobacco control efforts have largely focused on smoking cessation, SLT cessation remains under-researched, particularly in the South Asian Region, where SLT use surpasses smoking^5^.

Globally, the control measures are primarily smoking-centered even though countries like India and Bangladesh bear a significant SLT burden^6-8^. In India, 21.4% of the population uses SLT, which is more than double the smoking prevalence of 10.7%^9^. SLT use in India is higher among men (men 29.6% vs women 12.8%) and predominantly concentrated in rural areas (urban 15.2% vs rural 24.6%)^10^. As per the National Family Health Survey (NFHS)-5 (2019–2021), the most common form of tobacco consumption among men is chewing paan masala or gutkha (14.6%), followed closely by cigarettes (13.2%) and khaini (12.1%)^11^. SLT is consumed in various forms in India, including khaini, paan masala with tobacco, gutka, and betel quid with tobacco^10^. Similarly, in Bangladesh, 20.6% of the adult population uses SLT, with 24.8% of them being women^12^. The socio-cultural acceptance of SLT in both countries presents a significant public health challenge^13^.

Despite legislative measures such as India’s ban on gutka^10^ and Bangladesh’s prohibition of SLT advertising^7^, the prevalence of SLT use remains disproportionately high in these countries. Several barriers hinder SLT cessation, with demographic factors and intentions to quit playing a critical role. Previous studies indicate that SLT use is more prevalent among older adults^13,14^, individuals with a lower level of education^9^, those in lower wealth quintiles, and those with greater exposure to SLT marketing^15^.

The Global Adult Tobacco Survey (GATS) is a nationally representative survey developed by WHO and CDC to monitor tobacco use and key control indicators among adults aged ≥15 years. Several countries have participated in two waves of this survey^16^. A study in Bangladesh comparing two GATS waves (2009 and 2017) reported that among adults who used SLT, those in the older age category (aged ≥65 years) and women, were less likely to intend to quit in the future, whereas those with higher education and the highest wealth quintiles were more likely to intend to quit in the future^17^.

Advice and support from healthcare providers is a well-recognized facilitator of tobacco cessation. Some studies have demonstrated a strong and consistent association between receiving advice from a healthcare professional and increased likelihood of attempting to quit smokeless tobacco^18,19^. This form of intervention is especially critical in countries with high tobacco use prevalence, as it provides adults who use SLT with both motivation and guidance to initiate cessation. Despite this, the coverage and effectiveness of such interventions for those who use SLT remain underexplored, particularly in low- and middle-income countries.

Despite the introduction of various tobacco control measures, enforcement remains a persistent challenge in both India and Bangladesh. While some studies have explored smoking cessation, there remains a limited body of evidence specifically focused on the facilitators and barriers to smokeless tobacco (SLT) cessation, particularly in the context of temporal changes across GATS waves.

This study aims to address this gap by systematically analyzing the key demographic, healthcare, and policy-related factors influencing SLT cessation in India and Bangladesh. It applies a new way of presenting multivariate decomposition analysis to not only assess whether these factors are associated with changes in quit attempts, but also to quantify the relative contribution of each factor to observed changes over time. The findings are expected to generate policy-relevant insights that can support the design of more targeted and effective SLT cessation interventions in both countries and in other similar contexts.

## METHODS

### Overview

This study involved a secondary analysis of GATS data from two waves conducted in India and Bangladesh. The total number of GATS participants which were aged ≥15 years, the proportion who used SLT currently or in the past 12 months (either daily or less than daily), and among them, the proportion who provided valid responses to questions on attempting to quit SLT (AQSLT) were compared. Only individuals with valid AQSLT responses were included in further analysis. While de-identified data were used, an ethical approval to conduct the analysis was granted by the Public Health Foundation of India Institutional Ethics Committee (Ref. TRC-IEC 479/21).

### Data

This study used data from two waves of GATS conducted in India (2009–2010 and 2016–2017) and Bangladesh (2009 and 2017). GATS uses a standardized interviewer-administered questionnaire and methodology across participating countries to ensure cross-country comparability in collecting self-reported data on both smoked and smokeless tobacco use, including prevalence, cessation behavior, and media messaging, making it a valuable tool for understanding patterns and determinants of SLT use at the population level^16,20^. The datasets are publicly accessible along with details of sampling procedures and data collection methods through the GTSS Data Portal (see Data Availability section).

### Outcome variable

The outcome variable, attempted quitting smokeless tobacco (AQSLT), was defined as a self-reported attempt to quit SLT use within the past 12 months, regardless of outcome. For adults currently using SLT at the time of survey, this was based on the question: ‘During the past 12 months, have you tried to stop using smokeless tobacco?’ with responses recorded as either ‘yes’ or ‘no’. Adults who used SLT in the past but not at the time of survey, and reported quitting less than a year ago, were also included and considered to have made a quit attempt.

### Explanatory independent variables

The explanatory variables included key demographic and socioeconomic characteristics: age (15–24, 25–34, 35–44, and ≥45 years), sex (male, female), residence (urban, rural), education level (re-categorized into four categories of no formal education, primary or lower including those with any formal education up to completing primary school, secondary or lower including those continued education into secondary or high school up to graduation from high school, and higher education including any formal education after high school), employment status (recategorized into two categories of employed including any type of employment and unemployed/other including unemployed, homemaker, student, retired, and other), and Wealth index (low, medium, high)^21^. Wealth index was originally created by GATS using principal component analysis (PCA) based on the respondent’s ownership of certain household items^20^.

### Smokeless tobacco-related independent variables

Several smokeless tobacco (SLT)-specific variables were included. Participants were asked whether a doctor or healthcare provider advised them to stop using SLT in the past 12 months (yes, no). Awareness of health warnings on SLT product packaging was also recorded as a dichotomous variable (yes, no).

Exposure to anti-SLT messaging was assessed across three channels: print media (newspapers, magazines, posters, billboards), digital media (TV, radio), and other sources (unspecified). Each was analyzed separately as a dichotomous variable (yes, no).

Exposure to pro-SLT messaging was assessed via multiple sources including traditional media (e.g. print, radio, cinema), public spaces (walls, transport, clothing), and (in Wave 2) the internet. These were combined into a single dichotomous variable indicating any pro-SLT exposure in the past 30 days (yes, no), due to overlap and low response frequencies for individual sources.

### Statistical analysis

Analyses were conducted using STATA version 17, with a significance level set at α=0.05. The datasets were reviewed for completeness, cleaned, and used to generate descriptive statistics such as frequencies and percentages.

Changes in: 1) the total number of participants, 2) the number and percentage of adults who used SLT currently or during last 12 months, and 3) the number and percentage of those who provided valid responses to the AQSLT question, were calculated. Relative changes (RCs) were also calculated to assess the size of change using the formula: [(Percentage in Wave 2 - Percentage in Wave 1)/Percentage in Wave 1] × 100. Z-tests were used to test for significance of the changes. Differences in sample composition across categories of explanatory variables between Wave 1 and Wave 2 were assessed using chi-squared tests and RC calculations.

Multivariate logistic regression models were used to identify factors associated with AQSLT in each wave, adjusting for all explanatory variables. Adjusted odds ratios (AORs) with 95% confidence intervals are reported. Sample weights provided by GATS were applied to account for complex survey design and ensure nationally representative estimates. Variance inflation factors (VIFs) were examined to assess multicollinearity among independent variables; no concerns were identified (all VIFs <10). RCs and z-tests were also applied to evaluate changes in AQSLT percentages within each category of explanatory variables between waves.

Finally, as the main analysis, a multivariate decomposition analysis based on logit models^21,22^ was conducted to examine the contribution of each factor to the overall change in AQSLT between the two waves. This analysis decomposes the change into three components:

- Endowment effect (E): change due to differences in sample characteristics (e.g. more rural respondents)
- Composition effect (C): change due to shifts in behaviors or attitudes within categories (e.g. rural respondents more likely to try quitting)
- Residual effect (R): unexplained variation not captured by E or C.

## RESULTS

### Sample overview

In India, the total GATS sample size increased from 69296 in Wave 1 to 74037 in Wave 2, representing an RC of 6.84% (p<0.001). In Bangladesh, the sample also grew significantly from 9629 in Wave 1 to 12783 in Wave 2 (RC=32.76%’ p<0.001) (Table 1).

**Table 1:** Assessing the change in characteristics of participants in samples of Wave 1 (2009–2010) to Wave 2 (2016–2017) in India and Bangladesh from the Global Adult Tobacco Survey (GATS)

*Characteristics*	*India*						*Bangladesh*					
	*Wave 1*		*Wave 2*		*RC%*	*p ^a^*	*Wave 1*		*Wave 2*		*RC%*	*p ^a^*
	*n*	*Percent of all participants (95% CI)*	*n*	*Percent of all participants (95% CI)*			*n*	*Percent of all participants (95% CI)*	*n*	*Percent of all participants (95% CI)*		
Total number of participants in GATS	69296	-	74037	-	6.84	**<0.001**	9629	-	12783	-	32.76	**<0.001**
Number of adults who used SLT currently or during last 12 months	17258	24.90 (24.53–25.28)	15480	20.91 (20.58–21.24)	-16.02	**<0.001**	2690	27.94 (26.89–29.01)	3119	24.40 (23.55–25.27)	-12.67	**<0.001**
Number of valid responses to AQSLT in the past 12 months Question	17161	24.76 (24.40–25.15)	15470	20.89 (20.57–21.23)	-15.63	**<0.001**	2681	27.84 (26.80–28.92)	3116	24.38 (23.55–25.25)	-12.43	**<0.001**
**Sample characteristics among those with AQSLT valid responses**												
***Explanatory/smoking-related independent variable***	***n of category***	***Percent of total (95% CI)***	***n of category***	***Percent of total (95% CI)***	***RC%***	***p ^b^***	***n of category***	***Percent of total (95% CI)***	***n of category***	***Percent of total (95 % CI)***	***RC%***	***p ^b^***
Total	17161	100	15470	100	0	-	2681	100	3116	100	0	-
**Age** (years)												
15–24	2094	12.20 (11.68–12.74)	1244	8.04 (7.60–8.50)	-34.10	**<0.001**	140	5.22 (4.40–6.16)	104	3.34 (2.73–4.04)	-36.02	**<0.001**
25–34	4576	26.67 (25.90–27.45)	3841	24.83 (24.05–25.63)	-6.90		516	19.25 (17.62–20.98)	569	18.26 (16.79–19.82)	-5.14	
35–44	4701	27.39 (26.62–28.19)	4188	27.07 (26.26–27.9)	-1.17		758	28.27 (26.30–30.36)	785	25.19 (23.46–27.02)	-10.89	
≥45	5790	33.74 (32.88–34.62)	6197	40.06 (39.07–41.07)	18.73		1267	47.26 (44.69–49.93)	1658	53.21 (50.68–55.83)	12.59	
**Sex**												
Female	6725	39.19 (38.26–40.14)	5675	36.68 (35.74–37.65)	-6.40	**<0.001**	1438	53.64 (50.90–56.48)	1949	62.55 (59.80–65.39)	16.62	**<0.001**
Male	10436	60.81 (59.65–61.99)	9795	63.32 (62.07–64.58)	4.13		1243	46.36 (43.82–49.01)	1167	37.45 (35.33–39.66)	-19.22	
**Residence**												
Urban	5033	29.33 (28.52–30.15)	3696	23.89 (23.13–24.67)	-18.55	**<0.001**	1161	43.30 (40.85–45.87)	1357	43.55 (41.26–45.93)	0.58	0.851
Rural	12128	70.67 (69.42–71.94)	11774	76.11 (74.74–77.50)	7.70		1520	56.70 (53.88–59.62)	1759	56.45 (53.84–59.15)	-0.44	
**Education level**												
No formal schooling	5799	33.90 (33.03–34.78)	4950	32.02 (31.13–32.92)	–5.55	**<0.001**	1463	55.15 (52.36–58.05)	1478	47.43 (45.04–49.91)	-14.00	**<0.001**
Primary (or lower)	4992	29.18 (28.38–30.00)	4686	30.31 (29.45–31.19)	3.87		715	26.95 (25.01–29.00)	953	30.58 (28.67–32.59)	13.47	
Secondary (or lower)	4395	25.69 (24.94–26.46)	4218	27.28 (26.47–28.12)	6.19		381	14.36 (12.96–15.58)	556	17.84 (16.39–19.39)	24.23	
Higher education	1922	11.23 (10.74–11.75)	1606	10.39 (9.89–10.91)	-7.48		94	3.54 (2.86–4.34)	129	4.14 (3.46–4.92)	16.95	
**Employment status**												
Unemployed/other	5903	34.40 (33.53–35.29)	4565	29.51 (28.66–30.38)	-14.22	**<0.001**	1427	53.23 (50.50–56.06)	1889	69.62 (57.92–63.42)	30.79	**<0.001**
Employed	11258	65.6 (64.40–66.83)	10905	70.49 (69.17–71.83)	7.45		1254	46.77 (44.22–49.44)	1227	39.38 (37.20–41.64)	-15.80	
**Wealth index**												
Low	10426	60.75 (59.9–61.93)	10352	66.92 (65.63–68.22)	10.16	**<0.001**	1364	50.88 (48.21–53.65)	1605	51.51 (49.02–54.09)	1.24	**0.006**
Medium	2669	15.56 (14.97–16.15)	2203	14.24 (13.65–14.85)	-8.48		490	18.28 (16.69–19.97)	651	20.89 (19.32–22.56)	14.28	
High	4066	23.69 (22.97–24.33)	2915	18.84 (18.17–19.54)	-20.47		827	30.85 (28.78–33.20)	860	27.60 (25.79–29.51)	-10.53	
**Advised by a doctor or healthcare provider to quit SLT in the past 12 months**												
No	15080	87.87 (86.48–89.29)	13279	85.84 (84.38–87.31)	-2.31	**<0.001**	2050	76.46 (73.19–79.85)	2857	91.69 (88.36–95.11)	19.92	**<0.001**
Yes	2081	12.13 (11.61–12.66)	2191	14.16 (13.58–14.77)	16.74		631	23.54 (21.74–25.45)	259	8.31 (7.33–9.39)	-64.7	
**Noticed any health warning on SLT products**												
No	6892	40.18 (39.24–41.14)	4734	30.61 (29.74–31.49)	-23.82	**<0.001**	2455	91.57 (87.98–95.27)	1311	42.07 (39.83–44.41)	-54.06	**<0.001**
yes	10260	59.82 (58.67–60.99)	10734	69.39 (68.09–70.72)	16.0		226	8.43 (7.37–9.60)	1805	57.93 (55.29–60.66)	587.19	
**Noticed any information on print media related to dangers of SLT**												
No	8992	65.48 (64.13–66.84)	8900	58.32 (57.11–59.54)	-10.93	**<0.001**	2416	90.12 (86.56–93.78)	2653	85.14 (81.93–88.44)	-5.53	**<0.001**
Yes	4741	34.52 (33.55–35.52)	6361	41.68 (40.66–42.72)	20.74		265	9.88 (8.73–11.15)	463	14.86 (13.54–16.28)	50.4	
**Noticed any information on digital media related to dangers of SLT**												
No	7539	54.22 (53.00–55.46)	7057	46.55 (45.47–47.65)	-14.15	**<0.001**	2017	75.23 (71.99-78.59)	2311	74.17 (71.17–77.25)	-1.41	0.351
Yes	6366	45.78 (44.66–46.92)	8103	53.45 (52.29–54.63)	16.75		664	24.77 (22.92–26.72)	805	25.83 (24.08–27.67)	4.28	
**Noticed any information anywhere else related to dangers of SLT**												
No	16680	97.24 (95.77–98.72)	15342	99.18 (97.62–100.79)	2.0	**<0.001**	2661	99.25 (95.52–103.1)	3095	99.33 (95.86–102.89)	0.08	0.744
Yes	474	2.76 (2.52–3.02)	127	0.82 (0.68–0.98)	-70.29		20	0.75 (0.46–1.15)	21	0.67 (0.42–1.03)	-10.67	
**Exposure to pro-SLT advertisements or signs**												
No	13862	80.82 (79.48–82.18)	11675	75.47 (74.11–76.86)	-6.62	**<0.001**	2255	84.11 (80.67–87.66)	2860	91.78 (88.45–95.21)	9.12	**<0.001**
Yes	3290	19.18 (18.53–19.85)	3794	24.53 (23.75–25.32)	27.89		426	15.89 (14.42–17.47)	256	8.22 (7.24–9.29)	-48.27	

RC%: relative change=[Wave 2(%) – Wave 1(%)/Wave 1(%)] × 100. AQSLT: attempted to quit smokeless tobacco (adults who currently used SLT and tried to stop smoking in the past 12 months).

a Z-test.

b Chi-squared test, for contingencies testing the differences in the characteristics of participants between Wave 1 and Wave 2. Analyses in Tables 1 and 3 were conducted to help understanding the contribution of background factors to changes in AQSLT in the multivariate decomposition analysis. Estimates reflect the analytical sample used for decomposition and may differ slightly from nationally weighted GATS percentages. p<0.05 statistically significant.

The prevalence of adults who used SLT currently or during the last 12 months declined in both countries. In India, it decreased from 24.90% in Wave 1 to 20.91% in Wave 2 (RC= -16.02%, p<0.001). In Bangladesh, it dropped from 27.94% to 24.40% (RC= -12.67%, p<0.001).

The number of adults with valid responses to the AQSLT question closely matched the total number of those who used SLT either currently or in the past 12 months across both waves, with minimal exclusions due to missing or refused responses. The rate of such exclusions was lower in Wave 2 for both countries.

### Sample characteristics differences between Wave 1 and Wave 2

In India, statistically significant shifts were observed across all sample characteristics and smokeless tobacco-related independent variables (p<0.001 for all). The largest relative change (RC= -34.10%) occurred in the youngest age group (15–24 years). A smaller decrease was noted in the 25–34 age group (RC= -6.90%), while the 35–44 group showed a minimal increase (RC= -1.17%). The ≥45 years age group was the only age category with an increase in proportion (RC=18.73%). Bangladesh experienced the same pattern of change within its age groups (Table 1).

In India, significant increases were seen in the proportions of respondents who reported noticing health warnings on SLT products, exposure to anti-SLT messages in print media, and exposure to such messages in digital media. Interestingly, there was also an increase in reported exposure to pro-SLT advertisements and promotional content.

In Bangladesh, differences in place of residence, exposure to anti-SLT information via digital media, and noticing SLT-related information through other unspecified sources were not statistically significant. Other sample characteristics and SLT-related variables did show significant changes between the two waves.

### Association between independent variables and AQSLT within Wave 1 and Wave 2

In India, all older age groups had significantly lower adjusted odds ratios (AORs) for AQSLT compared to the youngest group (aged 15–24 years), with p<0.003 across all categories (Table 2). In Bangladesh, same pattern of AORs was observed; however, they did not reach statistical significance.

**Table 2:** Associated factors of attempts to quit smokeless tobacco (AQSLT) across two waves of Global Adult Tobacco Survey (GATS) in India (2009–2010, N=12434; 2016–2017, N=15069) and Bangladesh (2009, N=2653; 2017, N=1993) based on multivariate logistic regression models adjusted for GATS weight and strata

*Independent factors*	*India*								*Bangladesh*							
	*Wave 1*				*Wave 2*				*Wave 1*				*Wave 2*			
	*AOR*	*p*	*95% CI*		*AOR*	*p*	*95% CI*		*AOR*	*p*	*95% CI*		*AOR*	*p*	*95 % CI*	
			Lower	Upper			Lower	Upper			Lower	Upper			Lower	Upper
**Age** (years)																
15–24 ®	1.00				1.00				1.00				1.00			
25–34	0.82	**0.003**	0.72	0.94	0.92	0.280	0.80	1.07	0.89	0.671	0.51	1.54	0.84	0.583	0.44	1.59
35–44	0.79	**0.001**	0.70	0.91	0.86	**0.044**	0.75	0.99	0.88	0.641	0.52	1.50	0.88	0.700	0.47	1.65
≥45	0.79	**<0.001**	0.69	0.90	0.80	**0.002**	0.69	0.92	0.60	0.058	0.35	1.02	0.80	0.480	0.43	1.49
**Gender**																
Female ®	1.00				1.00				1.00				1.00			
Male	0.99	0.876	0.89	1.10	0.97	0.503	0.88	1.06	0.68	**0.037**	0.48	0.98	0.77	0.184	0.52	1.13
**Residence**																
Urban	1.00				1.00				1.00				1.00			
Rural	0.94	0.159	0.86	1.02	0.84	**<0.001**	0.77	0.91	0.73	0.015	0.57	0.94	0.800	0.100	0.61	1.04
**Education level**																
No formal education ®	1.00				1.00				1.00				1.00			
Primary	0.93	0.197	0.83	1.04	1.06	0.261	0.96	1.17	1.05	0.760	0.79	1.39	1.01	0.946	0.77	1.33
Secondary	1.04	0.552	0.92	1.17	1.21	**<0.001**	1.09	1.34	1.44	0.058	0.99	2.10	1.00	0.981	0.71	1.42
Higher education	1.09	0.273	0.94	1.27	1.32	**<0.001**	1.15	1.53	1.22	0. 558	0.63	2.38	1.34	0.422	0.66	2.72
**Employment status**																
Unemployed/other ®	1.00				1.00				1.00				1.00			
Employed	1.09	0.084	0.99	1.21	1.09	0.071	0.99	1.20	1.22	0.294	0.84	1.76	0.76	0.176	0.50	1.13
**Wealth index**																
Low ®	1.00				1.00				1.00				1.00			
Medium	0.98	0.687	0.88	1.09	1.15	**0.009**	1.04	1.28	0.78	0.134	0.56	1.08	0.91	0.526	0.67	1.23
High	0.83	**<0.001**	0.75	0.92	1.00	0.928	0.90	1.12	0.82	0.192	0.61	1.10	1.18	0.275	0.87	1.60
**Advised by a doctor or healthcare provider on quitting SLT in the past 12 months**																
No ®	1.00				1.00				1.00				1.00			
Yes	2.50	**<0.001**	2.24	2.79	2.53	**<0.001**	2.30	2.78	2.16	**<0.001**	1.64	2.83	1.02	0.951	0.62	1.67
**Noticed any health warning on SLT products**																
No ®	1.00				1.00				1.00				1.00			
Yes	1.41	**<0.001**	1.29	1.55	1.15	**0.002**	1.05	1.25	1.26	0.259	0.84	1.90	1.33	**0.020**	1.04	1.69
**Noticed any information on print media related to dangers of SLT**																
No ®	1.00				1.00				1.00				1.00			
Yes	1.32	**<0.001**	1.20	1.45	1.29	**<0.001**	1.18	1.41	0.83	0.409	0.54	1.28	1.99	**<0.001**	1.40	2.82
**Noticed any information on digital media related to dangers of SLT**																
No ®	1.00				1.00				1.00				1.00			
Yes	1.12	**0.017**	1.02	1.22	1.08	0.098	0.99	1.18	1.75	**<0.001**	1.31	2.34	0.66	**0.007**	0.49	0.89
**Noticed any information on anywhere else related to dangers of SLT**																
No ®	1.00				1.00				1.00				1.00			
Yes	1.07	0.547	0.86	1.32	1.56	**0.017**	1.08	2.25	2.95	0.102	0.81	10.77	1.66	0.489	0.40	6.95
**Exposure to pro-smokeless tobacco advertisement**																
No ®	1.00				1.00				1.00				1.00			
Yes	1.18	**0.001**	1.07	1.29	1.13	**0.006**	1.03	1.23	2.40	**<0.001**	1.78	3.26	1.29	0.245	0.84	2.00

SLT: smokeless tobacco, AOR: adjusted odds ratio. Individuals with missing responses for any variable included in this model were excluded from the analysis. ® Reference categories. p<0.05 statistically significant.

Male participants had a significantly lower likelihood of attempting to quit only in Wave 1 in Bangladesh (AOR=0.68; 95% CI: 0.48–0.98, p=0.037). Rural residence was associated with significantly lower odds of AQSLT in Wave 2 of India (AOR=0.84; 95% CI: 0.77–0.91, p<0.001) and Wave 1 of Bangladesh (AOR=0.73; 95% CI: 0.57–0.94, p=0.013). In Wave 2 of India, individuals with secondary or higher level of education had significantly greater odds of AQSLT compared to those with no formal schooling and primary education. Also, those in the medium Wealth index category were more likely to AQSLT than those in both low and high wealth categories.

Receiving advice from a healthcare provider to quit SLT within the past 12 months was strongly associated with AQSLT, with individuals who received such advice being more than twice as likely to report a quit attempt in Wave 1 (AOR=2.50; 95% CI: 2.24–2.79) and Wave 2 of India (AOR=2.53; 95% CI: 2.30–2.78) with a p<0.001 for both and Wave 1 of Bangladesh (AOR=2.16; 95% CI: 1.64–2.84, p<0.001).

Noticing health warnings on SLT product packaging and anti-SLT messages in print media was positively associated with AQSLT in both waves in India, and in Wave 2 in Bangladesh. Exposure to digital media warnings was significantly associated with higher odds of AQSLT in Wave 1 of both countries. However, this association disappeared in Wave 2 of India and reversed in Wave 2 of Bangladesh (AOR=0.66; 95% CI: 0.49–0.89, p=0.007).

Noticing SLT-related warnings from other unspecified sources was significantly associated with AQSLT only in Wave 2 of India (AOR=1.56; 95% CI: 1.08–2.25, p=0.017), at a time when digital media-specific warnings no longer showed a significant effect. This may point to the growing influence of broader or less traditional media channels not captured in media specific questions of GATS questionnaire.

Interestingly, exposure to pro-SLT advertisements was also associated with a higher likelihood of AQSLT in both waves of India and in Wave 1 of Bangladesh, a counterintuitive finding that may reflect increased awareness or cognitive dissonance rather than promotion-induced behavior.

### Shifts in AQS in total and within each category of independent variables from Wave 1 to Wave 2

Both India (RC=0.66%) and Bangladesh (RC=5.79%) experienced small, non-significant increases in overall AQSLT prevalence between Wave 1 and Wave 2 (Table 3).

**Table 3:** Assessing the change in prevalence of attempts to quit smokeless tobacco (AQSLT) in total and by explanatory and SLT-related independent variables in the past 12 months among adults who currently smoked in India and Bangladesh from Global Adult Tobacco Survey (GATS) Wave 1 (2009–2010) to Wave 2 (2016–2017)

*Explanatory/smoking-related independent variable*	*India*						*Bangladesh*					
	*Wave 1*		*Wave 2*		*RC% of AQSLT*	p* (AQSLT change)	*Wave 1*		*Wave 2*		*RC% of AQSLT*	p* (AQSLT change)
	*n of AQSLT*	*Percent in category (95% CI)*	*n of AQSLT*	*Percent in category (95% CI)*			*n of AQSLT*	*Percent in category (95% CI)*	*n of AQSLT*	*Percent in category (95 % CI)*		
**Total: Individuals who attempted to quit SLT in the past 12 months**	5104	29.74 (28.93–30.57)	4631	29.94 (29.08–30.81)	0.67	0.704	745	27.79 (25.83–29.86)	916	29.40 (27.52–31.36)	5.79	0.177
**Age** (years)												
15–24	723	34.53 (30.06–37.14)	410	32.96 (29.84–36.31)	–4.76	0.352	49	35.00 (25.89–42.67)	28	26.92 (17.89–38.91)	-23.14	0.180
25–34	1414	30.90 (29.31–32.55)	1231	32.05 (30.28–33.89)	3.72	0.258	169	32.75 (28.00–38.08)	171	30.05 (25.72–34.91)	-8.23	0.337
35–44	1383	29.42 (27.89–31.01)	1279	30.54 (28.89–32.26)	3.81	0.250	231	30.47 (26.67–34.67)	246	31.34 (27.54–35.51)	2.62	0.407
≥45	1584	27.36 (26.03–28.74)	1711	27.61 (26.32–28.95)	0.914	0.757	296	23.36 (20.78–26.18)	471	28.41 (25.90–31.09)	21.37	**0.002**
**Sex**												
Female	1828	27.18 (25.95–28.46)	1543	27.19 (25.85–28.58)	0.04	0.992	336	23.37 (20.93–26.00)	289	14.83 (13.17–16.64)	-8.15	**0.020**
Male	3276	31.39 (30.33–32.49)	3088	31.53 (30.42–32.66)	0.45	0.834	409	32.90 (29.79–36.25)	627	53.73 (49.60–58.1)	13.38	0.204
**Residence**												
Urban	1632	32.43 (30.87–34.04)	1289	34.88 (33.00–36.83)	7.55	**0.016**	366	31.52 (28.38–34.93)	456	33.6 (30.59–36.83)	6.67	0.267
Rural	3472	28.63 (27.68–29.60)	3342	28.38 (27.43–29.36)	–0.87	0.674	379	24.93 (22.49–27.58)	460	26.15 (23.82–28.65)	5.22	0.424
**Education level**												
No formal schooling	1511	26.06 (24.76–27.40)	1236	24.97 (23.60–26.40)	–4.18	0.197	373	25.50 (22.97–28.22)	406	27.47 (24.86–30.28)	7.84	0.267
Primary (or lower)	1438	28.81 (27.34–30.33)	1373	29.30 (27.77–30.89)	1.70	0.596	205	28.67 (24.88–32.88)	268	28.12 (24.86–31.70)	-2.09	0.803
Secondary (or lower)	1369	33.42 (31.74–35.18)	1420	33.67 (31.94–35.46)	0.75	0.810	133	34.91 (29.23–41.37)	191	34.35 (29.65–39.58)	-1.43	0.857
Higher education	673	35.02 (32.42–37.76)	599	37.30 (34.37–40.41)	6.51	0.159	34	36.17 (25.05–30.55)	51	39.53 (29.44–51.98)	9.12	0.610
**Employment status**												
Unemployed/other	1621	27.46 (26.14–28.83)	1245	27.27 (25.78–28.83)	–0.69	0.834	395	27.68 (25.02–30.55)	592	31.34 (28.87–33.97)	13.00	**0.023**
Employed	3483	30.94 (29.92–31.98)	3386	31.05 (30.01–32.11)	0.36	0.857	350	27.91 (25.06–30.99)	324	26.41 (23.61–29.44)	-5.38	0.401
**Wealth index**												
Low	2995	28.73 (27.71–29.77)	2882	27.84 (26.83–28.88)	–3.10	0.156	360	26.39 (23.74–29.27)	433	26.98 (24.50–29.64)	2.27	0.719
Medium	861	32.26 (30.14–34.49)	762	34.59 (32.18–37.13)	7.22	0.085	123	25.10 (20.86–29.95)	185	28.42 (24.47–32.82)	13.15	0.211
High	1248	30.69 (29.01–32.44)	987	33.86 (31.78–36.04)	10.33	**0.005**	262	31.68 (27.96–35.76)	298	34.65 (30.83–38.82)	9.46	0.194
**Advised by a doctor or healthcare provider to quit SLT in the past 12 months**												
No	4115	27.29 (26.46–28.13)	3570	26.88 (26.01–27.78)	-1.50	0.447	483	23.56 (21.51–25.76)	823	28.81 (26.87–30.84)	22.03	**<0.001**
Yes	989	47.53 (44.61–50.58	1061	48.43 (45.56–51.43)	1.89	0.555	262	41.52 (36.65–46.87)	93	35.91 (28.98–43.99)	-13.49	0.121
**Noticed any health warning on SLT products**												
No	1652	23.97 (22.83–25.15)	1171	24.74 (23.34–26.19)	3.21	0.342	656	26.72 (24.72–28.85)	352	26.85 (24.12–29.81)	0.37	0.928
Yes	3447	33.60 (32.48–34.74)	3460	32.23 (31.17–33.33)	-4.08	**0.036**	89	39.38 (31.63–48.46)	564	31.25 (28.72–33.94)	-20.81	**0.013**
**Noticed any information on print media related to dangers of SLT**												
No	2469	27.46 (26.39–28.56)	2283	25.65 (24.61–26.73)	-6.59	**0.006**	645	26.7 (24.68–28.84)	726	27.37 (25.41–29.43)	2.62	0.596
Yes	1800	37.97 (36.23–39.76)	2310	36.32 (34.85–37.83)	-4.35	0.075	100	37.74 (30.7–45.9)	190	41.04 (35.41–47.30)	8.75	0.379
**Noticed any information on digital media related to dangers of SLT**												
No	2055	27.26 (26.09–28.46)	1804	25.56 (24.40–26.77)	-6.24	**0.020**	496	24.59 (22.47–26.85)	657	28.43 (26.30–30.69)	15.45	**0.004**
Yes	2197	34.51 (33.08–35.99)	2770	34.18 (32.92–35.48)	-0.96	0.682	249	37.5 (32.99–42.46)	259	32.17 (28.37–36.34)	-14.13	**0.032**
**Noticed any information anywhere else related to dangers of SLT**												
No	4923	29.51 (28.70–30.35)	4576	29.83 (28.97–30.70)	1.08	0.542	734	27.58 (25.62–29.65)	905	29.24 (27.37–31.21)	5.80	0.164
Yes	178	37.55 (32.24–43.49)	55	43.31 (32.62–56.37)	15.34	0.238	11	55.00 (27.46–98.41)	11	52.38 (26.15–93.72)	8.00	0.865
**Exposure to pro-SLT advertisement**												
No	3901	28.14 (27.27–29.04)	3287	28.15 (27.20–29.13)	0.04	0.984	557	24.70 (22.69–26.84)	809	28.29 (26.37–30.30)	14.53	**0.004**
Yes	1198	36.41 (34.38–38.54)	1344	35.42 (33.56–37.37)	-2.72	0.384	188	44.13 (38.05–50.91)	107	41.80 (34.25–50.51)	-5.28	0.548

RC%: relative change=[Wave 2(%) – Wave 1(%)/Wave 1(%)] × 100. AQSLT: attempted to quit smokeless tobacco (adults who currently used smokeless tobacco and tried to stop smoking in the past 12 months).

* p-values are obtained using z-tests, testing the difference in percentages between Wave 1 and Wave 2 of each row. Analyses in Tables 1 and 3 were conducted to help understanding the contribution of background factors to changes in AQSLT in the multivariate decomposition analysis. Estimates reflect the analytical sample used for decomposition and may differ slightly from nationally weighted GATS percentages. Statistically significant p<0.05.

Among the explanatory independent variables, India showed two significant increases in AQSLT: among urban residents (RC=7.55%, p=0.016) and individuals in the high wealth index category (RC=10.33%, p=0.005).

In Bangladesh, a significant increase in AQSLT was observed in the ≥45 years age group, rising from 23.36% in Wave 1 to 28.41% in Wave 2 (RC=21.37%, p=0.002). Bangladeshi females showed a significant decline in AQSLT, dropping from 23.37% to 14.83% (RC= -8.15%, p=0.020). A significant increase was also seen among the unemployed/other group in Bangladesh (RC=13.00%, p=0.023).

Among SLT-related variables, AQSLT prevalence remained stable in India for both those who did and did not receive professional advice to quit. However, in Bangladesh, a significant increase was found among those who did not receive advice, from 23.56% to 28.81% (RC=22.03%, p<0.001). Interestingly, AQSLT among those who received such advice declined, although the change was not statistically significant.

AQSLT rates decreased significantly among those who noticed health warnings on SLT products in both India (RC= -4.08%, p=0.036) and Bangladesh (RC= -20.81%, p=0.013). Similarly, a significant decrease was observed in Bangladesh among those who reported exposure to SLT-related messages via digital media (RC= -14.13%, p=0.032).

In terms of exposure to pro-SLT advertising, Bangladesh showed a significant increase in AQSLT among those not exposed to such advertisements from 24.70% in Wave 1 to 28.29% in Wave 2 (RC=14.53%, p=0.004).

### Overview of relative contribution of predictors to change in AQSLT from Wave 1 to Wave 2

The first section of Table 4 presents the relative contributions of the endowment effect (E), composition effect (C), and residual effect (R) to changes in AQSLT prevalence between Wave 1 and Wave 2 in India and Bangladesh. None of the components showed statistically significant total contributions. However, the E and C components were further examined, as it remained possible that significant contributions by specific factors, either due to changes in sample characteristics (E) or shifts in behaviors within specific categories (C), happened but were offset by opposing effects elsewhere. This was particularly relevant given the overall non-significant change in AQSLT prevalence (Table 3).

**Table 4:** Relative contribution of predictors to the change in attempts to quit smokeless tobacco (AQSLT) between Wave 1 (2009–2010) and Wave 2 (2016–2017) of Global Adult Tobacco Survey (GATS) in India (N=32631) and Bangladesh (N=5797) using multivariate decomposition analysis

	*India*					*Bangladesh*				
	*Coef.*	*p*	*95% C*		Percent*	*Coef.*	*p*	*95% C*		Percent*
			*Lower*	*Upper*				*Lower*	*Upper*	
E	-0.00248	0.283	-0.00700	0.00204	26.52	0.0145	0.178	-0.0066	0.0356	110.28
C	-0.00686	0.248	-0.01850	0.00477	73.48	-0.0014	0.931	-0.0321	0.0294	-10.28
R	-0.00934	0.087	-0.02004	0.00136		0.0132	0.256	-0.0096	0.0359	
**Endowment effect (E)**										
**Age** (years)										
15–24										
25–34	0.00040	0.297	-0.00035	0.00115	-4.26	-0.0004	0.428	-0.0015	0.0006	-3.28
35–44	0.00029	0.066	-0.00002	0.00059	-3.06	-0.0017	0.236	-0.0044	0.0011	-12.55
≥45	-0.00365	**0.007**	-0.00632	-0.00198	39.08	0.0028	0.313	-0.0026	0.0083	21.31
**Gender**										
Female										
Male	0.00006	0.503	-0.00112	0.00024	-0.66	0.0078	**0.002**	0.0029	0.0126	58.91
**Residence**										
Urban										
Rural	-0.00309	**0.001**	-0.00497	-0.00121	33.07	0.0001	**0.003**	0.00003	0.0001	0.63
**Education level**										
No education										
Primary (or less)	-0.00007	0.264	-0.00018	0.00005	0.72	0.0002	0.825	-0.0012	0.0015	1.17
Secondary (or less)	-0.00112	**0.002**	-0.00181	-0.00043	12.00	0.0017	**0.045**	0.00004	0.0033	12.59
Higher education	-0.00208	**0.001**	-0.00332	-0.00085	22.31	0.0004	0.114	-0.0001	0.0009	3.83
**Employment status**										
Unemployed/other										
Employed	0.00059	0.079	-0.00007	0.00126	-6.36	0.0015	0.433	-0.0023	0.0054	11.69
**Wealth index**										
Low										
Medium	-0.00108	**0.019**	-0.00199	-0.00017	11.61	-0.00003	0.960	-0.0012	0.0011	-0.22
High	-0.00010	0.928	-0.00226	0.00206	1.06	-0.0010	0.131	-0.0024	0.0003	-7.90
**Advised by a doctor or healthcare provider on quitting SLT in the past 12 months**										
No										
Yes	0.00278	**<0.001**	0.00205	0.00351	-29.76	-0.0056	0.216	-0.0144	0.0033	-42.37
**Noticed any health warning on SLT products**										
No										
Yes	0.00212	**0.003**	0.00072	0.00352	-22.71	0.0095	0.256	-0.0069	0.0259	72.22
**Noticed any information on print media related to dangers of SLT**										
No										
Yes	0.00289	**<0.001**	0.00172	0.00407	-30.98	0.0056	**<0.001**	0.0032	0.0079	42.34
**Noticed any information on digital media related to dangers of SLT**										
No										
Yes	0.00103	0.082	-0.00013	0.00219	-11.05	-0.0003	0.064	-0.0006	0.00002	-2.27
**Noticed any information anywhere else related to dangers of SLT**										
No										
Yes	-0.00191	0.053	-0.00384	0.00003	20.42	-0.0001	0.218	-0.0002	0.0001	-0.66
**Exposure to pro-SLT advertisements**										
No										
Yes	0.00046	0.011	**0.00011**	0.00081	-4.93	-0.0058	**0.005**	-0.0092	-0.0018	-44.35
**Total**					26.52					110.28
**Coefficient effect (C)**										
**Age** (years) 15–24										
25–34	0.00681	0.247	-0.00471	0.01834	-72.95	-0.0029	0.944	-0.0836	0.0779	-21.88
35–44	0.00504	0.402	-0.00674	0.01681	-53.91	-0.0069	0.944	-0.2014	0.1875	-52.75
≥45	0.00091	0.886	-0.01153	0.01336	-9.77	-0.0178	0.944	-0.5159	0.4804	-135.16
**Gender**										
Female										
Male	-0.00329	0.735	-0.02234	0.01577	35.19	0.0057	0.945	-0.155	0.1667	43.45
**Residence**										
Urban										
Rural	-0.01613	0.063	-0.03314	0.00089	172.62	0.0012	0.945	-0.0318	0.0341	8.78
**Education level**										
No education										
Primary (or less)	0.00866	0.097	-0.00156	0.01888	-92.73	0.0009	0.944	-0.0243	0.0261	6.82
Secondary (or less)	0.00990	0.069	-0.00075	0.02056	-106.01	0.0006	0.944	-0.0173	0.0186	4.88
Higher education	0.00601	0.069	-0.00046	0.01249	-64.37	0.0001	0.947	-0.0026	0.0028	0.70
**Employment status**										
Unemployed/other										
Employed	-0.00021	0.983	-0.02002	0.01959	2.28	0.0043	0.944	-0.1145	0.1231	32.41
**Wealth index**										
Low										
Medium	0.00635	**0.040**	0.00028	0.01242	-68.01	-0.0015	0.944	-0.0448	0.0417	-11.75
High	0.01249	**0.016**	0.00231	0.02268	-133.75	-0.0020	0.944	-0.0573	0.0533	-15.02
**Advised by a doctor or healthcare provider on quitting SLT in the past 12 months**										
No										
Yes	0.00034	0.860	-0.00346	0.00414	-3.66	0.0075	0.944	-0.2043	0.2194	57.30
**Noticed any health warning on SLT products**										
No										
Yes	-0.02714	**0.007**	-0.04679	-0.00749	290.54	0.0004	0.943	-0.0094	0.0101	2.72
**Noticed any information on print media related to dangers of SLT**										
No										
Yes	-0.00163	0.750	-0.01164	0.00839	17.43	-0.0030	0.944	-0.0869	0.0809	-22.78
**Noticed any information on digital media related to dangers of SLT**										
No										
Yes	-0.00329	0.600	-0.01559	0.00900	35.27	0.0061	0.944	-0.1651	0.1774	46.53
**Noticed any information anywhere else related to dangers of SLT**										
No										
Yes	0.00253	0.076	-0.00026	0.00531	-27.03	0.0001	0.945	-0.0029	0.0031	0.80
**Exposure to pro-SLT advertisements**										
No										
Yes	-0.00213	0.504	-0.00836	0.00410	22.75	0.0025	0.944	-0.0675	0.0725	18.92
**Total**					73.48					-10.28

Coef: coefficient.

* Percentage contribution. E: endowment effect. C: contribution effect. R: residual effect. Statistically significant p<0.05.

### Endowment effect: contributions to AQSLT change by change in sample characteristics

Despite significant differences in age distribution from Wave 1 to Wave 2 within both countries (Table 1), the multivariate decomposition revealed that the only significant contribution was made by the increase in the ≥45 years age group, which had a significant negative effect on the total AQSLT in India (percent contribution=39.08%, p=0.007) (Table 4). In terms of sex, a significant contribution was observed in Bangladesh, where the lower percentage of males in Wave 2 had a positive effect, equal to 58.91% of the total AQSLT change (p=0.002). For residence, the decreased proportion of urban residents in Wave 2 of India negatively influenced AQSLT change (33.07%, p<0.001). In contrast, a modest but significant positive contribution of 0.63% was seen in Bangladesh related to change in residence (p=0.003).

Significant endowment contributions to the total AQSLT were also observed within education categories of both countries and Wealth index of India.

In India, the increase in the proportion of individuals receiving quitting advice from doctors or health professionals from Wave 1 to Wave 2, and the increase in those noticing health warnings on SLT products, both had significant positive contributions towards total AQSLT change, accounting for 29.76% (p<0.001) and 22.71% (p=0.003) of total change, respectively. However, the change proportion of participant in neither of these factors had significant contribution in Bangladesh.

In terms of media exposure, the contribution to AQSLT change from Wave 1 to Wave 2 in India was only significant in those who noticed information related to danger of SLT in print media, which was positive in both India (30.98%, p<0.001) and Bangladesh (42.34%, p<0.001).

Table 2 presented the interesting fact that those exposed to pro-SLT advertisements and promotions were more like to AQSLT. This was resonated in endowment effect as in India, increased exposure to these advertisements from Wave 1 to Wave 2 had a significant positive contribution to total AQSLT change (4.93%, p=0.011), while in Bangladesh, decreased exposure resulted in a significant negative contribution (44.35%, p=0.005) after adjustments were made.

### Composition effect: changes in respondent attitudes on AQSLT from Wave 1 to Wave 2

The only two variables with a significant composition effect on total AQSLT change in India were the Wealth index and noticing health warnings on SLT products (Table 4). Individuals in the medium and high Wealth index categories showed increased AQSLT rates between waves, which contributed positively to the overall change – 68.01% (p=0.040) for the medium and 133.75% (p=0.016) for the high Wealth index group. In contrast, a significant decline in AQSLT among those who noticed health warnings on SLT products contributed negatively. This single factor accounted for a large negative contribution of 290.54% (p=0.007) to the total AQSLT change. In Bangladesh, no single variable had a significant composition effect, with most p-values being close to 1.

## DISCUSSION

This study provides a comparative analysis of quit attempts among adults using SLT in India and Bangladesh, using two waves of nationally representative GATS data. Despite a significant reduction in the overall prevalence of SLT use between waves, the percentage of AQSLT showed only small, non-significant increases in both countries from Wave 1 to Wave 2.

The Longitudinal Aging Study in India (LASI) reported a prevalence of current SLT use of 17.2% among older adults, which is substantially lower than the proportions observed in the GATS data used in this study^23^. This discrepancy may reflect differences in sampling design, self-reporting accuracy, or broader definitions of SLT use across surveys, but it also highlights the continued high burden of SLT use among Indian adults, particularly beyond middle age^23^.

The finding that females constitute a large proportion of SLT users in Bangladesh contrasts with tobacco smoking patterns, where, based on previous studies using the same GATS data, females made up only a small fraction of adults who smoked tobacco^21^.

The pattern of change within the age groups in India suggests that the overall decline in the proportion of adults who used SLT currently or during the last 12 months in Wave 2 compared to Wave 1 of India might have primarily been driven by reduced initiation among younger participants, rather than cessation among older age groups. Older adults consistently exhibited lower odds of AQSLT, particularly in India, where the endowment effect of an increased ≥45 years age group negatively impacted total AQSLT. This aligns with existing literature indicating that older adults using SLT are less likely to attempt quitting and may require targeted interventions to overcome entrenched behaviours^24^.

Education was positively associated with quit attempts, particularly in Wave 2 of India, and decomposition analysis supported a positive contribution of higher level of education to AQSLT. These findings corroborate earlier studies suggesting higher level of education enhances health literacy and readiness to quit SLT^25,26^.

In Bangladesh, significant gender disparities emerged, with a decline in AQSLT among females despite an increasing share of women in the SLT-using population. This suggests a need for gender-sensitive interventions. While previous data from NFHS-4 showed Indian women were less likely to intend or succeed in quitting SLT^27^, the current findings indicate that in Bangladesh, this trend may be even more pronounced.

Advice from healthcare providers was a strong and consistent predictor of AQSLT in both waves of India and Wave 1 of Bangladesh. However, the unchanged quit attempt rates among those who did and did not receive advice in India suggest gaps in either the frequency or quality of cessation support. As previous studies suggest, routine integration of tobacco use history and quit support in primary care can play a transformative role^28^.

Exposure to anti-SLT warnings had mixed effects. While associated with higher odds of quit attempts in some groups (e.g. print media in India), decomposition analysis showed a negative contribution from those who noticed SLT warnings on packaging – especially in India. This may indicate that warning labels are losing their impact over time, possibly due to poor design, cultural misinterpretation, or competing product packaging^29^. These findings are consistent with studies suggesting the need for more impactful, culturally appropriate, and regularly updated warnings^30^.

The most unexpected result was that exposure to pro-SLT advertising was associated with higher AQSLT odds in both waves of India and Wave 1 of Bangladesh. That exposure change had positive association with AQSLT in India but negative one in Bangladesh. While counterintuitive, this may reflect heightened awareness or cognitive dissonance among those exposed to conflicting messages^31^. It also underscores the complex role of media and the need to monitor not just the presence but also the content and framing of pro- and anti-SLT messaging^31^.

Despite progress in policies for tobacco control in both countries, including expanded tobacco control laws and increase in awareness campaigns, cessation support for SLT remains limited. Treatment services, especially those tailored for SLT cessation, are scarce and not evidence based. This gap may partly explain the disconnection between reduced prevalence and stagnant quit attempts. Tobacco control programs must address the serious misconceptions that SLT is harmless or helpful for health issues, particularly in rural and underserved populations^7,31^.

### Strengths and limitations

This study’s key strength lies in the use of multivariate decomposition analysis, which allowed for a nuanced examination of both demographic shifts and behavioral change. The large, nationally representative GATS data add to the generalizability of the findings across both countries.

Limitations include the cross-sectional nature of GATS data, precluding causal inference. Composite media exposure variables may have masked specific media effects. Self-reported quit attempts are also susceptible to recall and social desirability bias. Despite the inclusion of a broad range of explanatory variables, the significant residual component (‘R’) in decomposition analysis of India likely reflects unmeasured influences on smoking cessation. Furthermore, GATS Wave 2 data predate the full-scale rollout of interventions like India’s mCessation program^32^, potentially underestimating the current policy impact.

### Policy and practice implications

Findings highlight the importance of strengthening cessation infrastructure and support systems. Revisiting SLT cessation advice program, culturally tailored public messaging, and improving enforcement against pro-SLT advertising could help support quit attempts. Warning labels require dynamic redesign and field testing to enhance relevance and impact.

### Future research

Further research could explore the effectiveness of different SLT cessation approaches, including digital and AI-based interventions. Qualitative studies targeting women and older adults that use SLT, and other marginalized groups, may uncover barriers and facilitators unique to these populations. Longitudinal data capturing transitions in SLT use and multiple quit attempts would offer deeper insights into sustained cessation trajectories.

## CONCLUSIONS

There has been a modest decline in the overall prevalence of SLT use between Wave 1 and Wave 2 of GATS in India and Bangladesh. However, the proportion of adults who attempted to quit SLT remained nearly unchanged, despite substantial tobacco control efforts during the same period. Several background and socioeconomic factors appear to be influential. Although the percentages of individuals receiving professional advice or noticing warning messages increased, multivariate analysis questions their effectiveness, especially regarding the warnings in SLT products. Future research should focus on evaluating how these interventions are implemented, exploring culturally tailored approaches, and identifying communication strategies that effectively motivate quit attempts among SLT users.

## Data Availability

The data supporting this research are publicly available from GTSS through CDC (About GTSS | Smoking & Tobacco Use | CDC) and WHO (Noncommunicable Disease Surveillance, Monitoring and Reporting) websites.
